# Book Review: Young Children's Foreign Language Anxiety: The Case of South Korea

**DOI:** 10.3389/fpsyg.2021.812325

**Published:** 2021-12-02

**Authors:** Chunling Cao

**Affiliations:** School of Translation Studies, Shandong University, Weihai, China

**Keywords:** English education, children's language acquisition, foreign language anxiety, positive psychology, task-based language teaching

It has been stipulated that the role of English as a lingua franca (ELF) is becoming ever more essential in both business and education, so learning English has been regarded as one of the most precious forms of social capital among children at earlier stages of their lives inasmuch as the fact that it can empower individuals to successfully operate locally, nationally, and internationally. Therefore, in such a process of language learning, the role of foreign language anxiety (FLA) becomes more indispensable because if it is not recognized or addressed at earlier stages, it can have “detrimental effects not only on a child's language acquisition but also on the child's overall psychosocial well-being” (p. 2). *Young Children's Foreign Language Anxiety: The Case of South Korean*, written by Jieun Kiaer, Jessica M. Morgan-Brown, and Naya Choi, is opportune in that little research has been conducted on FLA in childhood language education, in general, and more specifically in the EFL context of South Korea, and it sheds light on our understanding of “the status quo of young children's FLA and the need to consider ways to decrease FLA in young children's English education” (p. 2), by offering possible solutions to construct more ethical environments where children can feel secure and discover language without feeling anxious. The other impetus behind this book is that “English language acquisition stands out as one of the most worrying social phenomena that young Koreans encounter” (p. 2) to the detriment of their academic, psychosocial and physical well-being, so when young children are taught in a free-anxiety context, not only do they fancy learning English, but also they will flourish and accomplish their learning targets.

Structurally, the book consists of eight chapters, organized into three sections. The first section encompasses three chapters, which specifically focus on the theoretical foundations for FLA. Focusing on foreign language classroom anxiety (FLCA), proposed by Horwitz et al. (1986), is the main concern of Chapter 2. The authors also discuss the psychological effects of FLA, by embarking on how positive psychology (PP) research and Dewaele and MacIntyre's (2016) research on foreign language enjoyment (FLE), have impacted perceptions of FLA/FLCA. Chapter 3 narrows the scope of FLA in young children, concentrating on the specific techniques that FLA showcases itself and multifarious variables that lead to FLA and FLCA in young children. The last chapter of this section specifically contextualizes FLA and FLCA in the South Korean context and extrapolates its implications to the Chinese, Japanese, and Taiwanese English language learning contexts, by elaborating on the psychological and emotional impacts of Korean early childhood English education.

The second section of this thought-provoking and updated monograph includes Chapters 5 and 6 which enumerates the problems and challenges that are related to FLA and early childhood English education. The authors in Chapter 5 brilliantly highlight the sources of FLA among young children and demystify the common myths (rote learning methods, immersion teaching methods, emphasis on “native speaker pronunciation” over communicative function), although widely accepted and practiced by the general public, have very little theoretical and empirical bases in application when it is applied to the young children's language education, leading the Korean authorities to give up offering early childhood English education. Chapter 6 presents case studies conducted on the Korean young children's foreign language anxiety (FLA), scrutinizing whether there could be any meaningful difference between kindergartens' children and English immersion institutions, concluding that family backgrounds, contextual factors, teacher-related factors can affect the level of FLA.

The third section of the book, encompassing Chapters 7 and 8, problematizes FLA in the Korean context and offers some practical solutions to the raised challenges. The authors adroitly propose the implementation of task-based language teaching (TBLT) and translanguaging pedagogical practice as innovative teaching practices in the English classroom. The final chapter, entitled “Towards a More Ethical Environment”, aims to move toward a learning environment by “focusing on the recognition, alleviation and prevention of FLA, as well as the encouraging of FLE” (p. 109). The authors cogently argue that creating a safe and anxiety-free learning environment is possible by “activating young children's minds rather than raising affective filters through FLA” (p. 3).

As an applied linguist and a practitioner, I feel that this insightful monograph is beneficial in that it provides plenty of fresh food for thoughts in terms of not only theory but also practice. In other words, it appropriately intermingles the germane and updated theories with empirical studies throughout the book. Moreover, the book draws on both qualitative and quantitative data from different sources which can enrich our understanding of the sources of FLA in the Korean context. What I also specifically like about the book is the validation of FLA in the Korean context that can also be utilized in other English language learning contexts such as China and Japan. Considering the applicability of the book, the book can inform parents, teachers, policymakers, and school administrations and provide them with some viable language classroom solutions. I reckon that since English language learning progressively and exponentially increases globally, understanding how to concomitantly teach children and safeguard their psychosocial well-being plays a crucial role.

## Author Contributions

The author confirms being the sole contributor of this work and has approved it for publication.

## Conflict of Interest

The author declares that the research was conducted in the absence of any commercial or financial relationships that could be construed as a potential conflict of interest.

## Publisher's Note

All claims expressed in this article are solely those of the authors and do not necessarily represent those of their affiliated organizations, or those of the publisher, the editors and the reviewers. Any product that may be evaluated in this article, or claim that may be made by its manufacturer, is not guaranteed or endorsed by the publisher.
